# Mr. Alfred Harold Godwin

**Published:** 1911-05-06

**Authors:** 


On April 24, Mr. Alfred Harold Godwin died at New Brighton, Cheshire, at the age of forty-four. He studied at London and at Liverpool, and took the conjoint diploma in 1893. He had been honorary surgeon to the Wallasey Cottage Hospital, but had since retired from practice.

